# Supplementary material for: Molecular-scale dynamics of light-induced spin cross-over in a two-dimensional layer
Source: Nat Commun. 2016 Jul 18;7:12212. doi: 10.1038/ncomms12212 (PMC4960305; doi:10.1038/ncomms12212)
Supplement: Supplementary Information — Supplementary Figures 1-7 [file ncomms12212-s1.pdf]

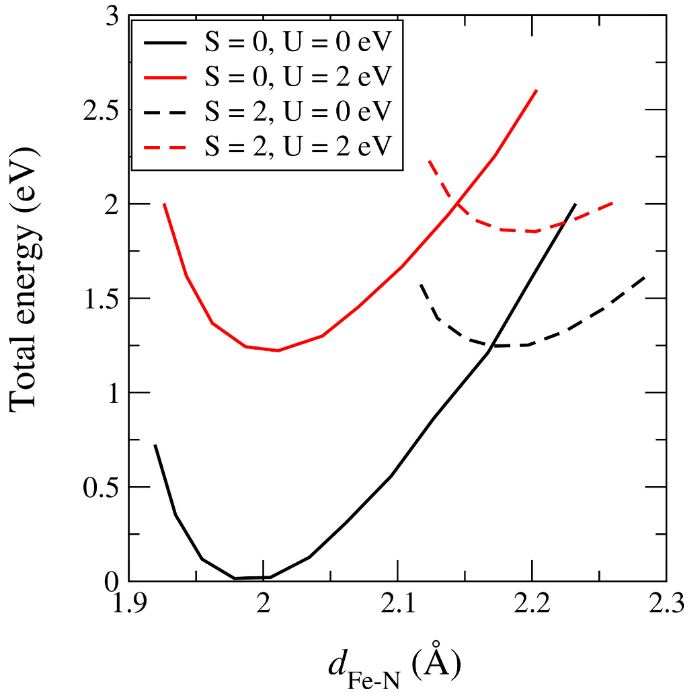

Supplementary Figure 1. **Total energy of the free molecule.** To understand the relation between LS and HS ground states, the total energy of the free molecule is plotted as a function of the Fe-N distance for both LS (solid lines) and HS (dashed lines) states obtained by DFT (black) and DFT+U (red) calculations. The spin state of SCO molecules is sensitive to the local environment of the transition metal ion, in particular the bond-lengths and the coordination number have an important role. For each calculation, the system is fully relaxed until the atomic forces are less than 0.001 eV/Å by fixing the (x, y, z) atomic position of Fe^{II} as well as the atomic positions of six surrounding N atoms in the z direction. Note that the total energy of the lowest energy configuration for LS states was set to be 0 eV as a reference. Since the lowest energy configuration is LS states, a constraint calculation was carried out in the case of HS states by fixing the spin moment of the molecule. The HS state thus obtained was found to be higher in energy of about 1.2 eV compared to LS state. We found that both the increase of $d_{\text{Fe-N}}$ and U favor the HS solution.

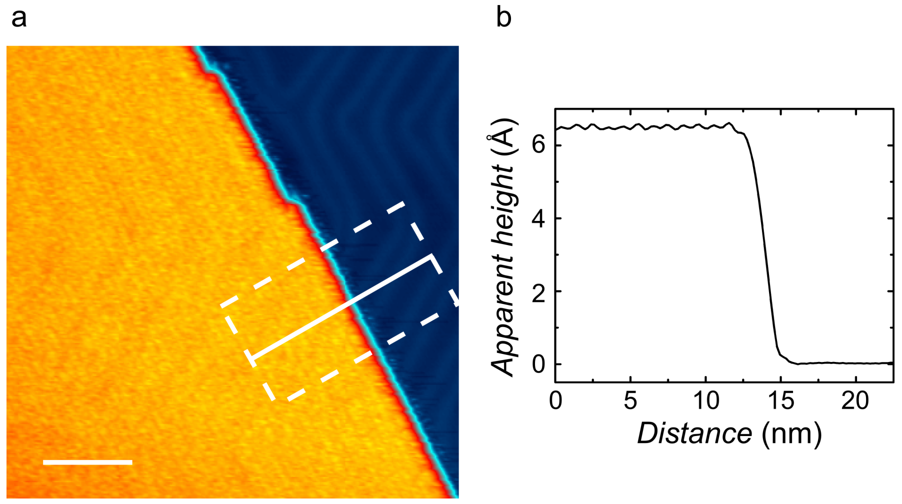

Supplementary Figure 2. **Submonolayer coverage.** (a) STM image of an island of compound **1** on Au(111) ($V = -1.5$ V, $I = 10$ pA). Bare reconstructed gold is visible on the upper right corner. The scale bar corresponds to 10 nm. (b) Mean profile across the border of the island (the dashed rectangle corresponds to the area of the mean profile). The apparent height of the island is 0.65 ± 0.01 nm which is compatible with the size of the molecule thus proving that compound **1** forms island of one monolayer height.

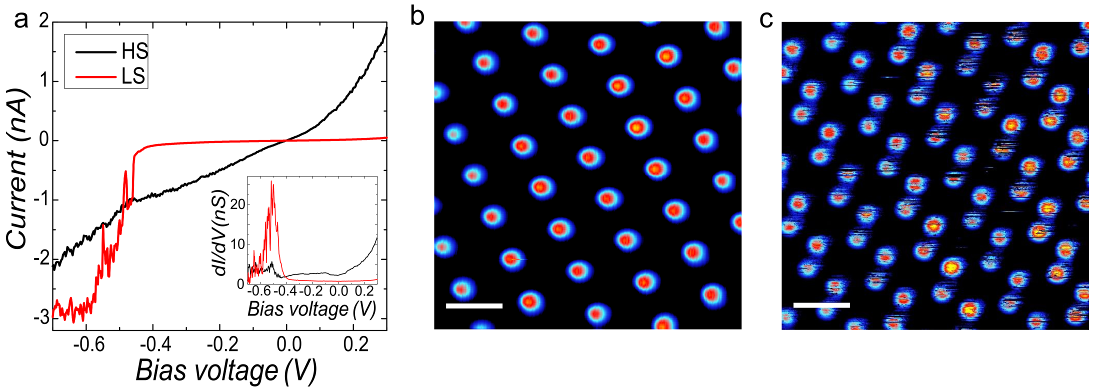

Supplementary Figure 3. **Electronic properties.** (a) I-V curves recorded at constant height between -0.7 V and $+0.3$ V over a LS molecule (red) and a HS molecule (black). The inset shows the differential conductance (dI/dV) recorded at the same locations with a lock-in amplifier (675 Hz modulation of 24 mV). The density of states is more important in positive bias for HS molecule and in negative bias for LS molecule, which is in good agreement with the DFT calculations that find $d_{x^2-y^2}$,d_{xy}-states as LUMO state for HS molecule and as HOMO state for LS molecule. Those empty and filled states are directly visible in constant height images recorded either in positive ($+0.3$ V) or negative bias voltages (-0.7 V). (b) Constant height image recorded at $+0.3$ V that shows increased absolute values of current on the HS molecules (similar to Figure 1b of the main text, $< I > = 50$ pA). (c) Constant height image of the same area than (b) recorded at -0.7 V that shows increased absolute values of current on the LS molecules ($< I > = 50$ pA). The scale bars in both images correspond to 2 nm.

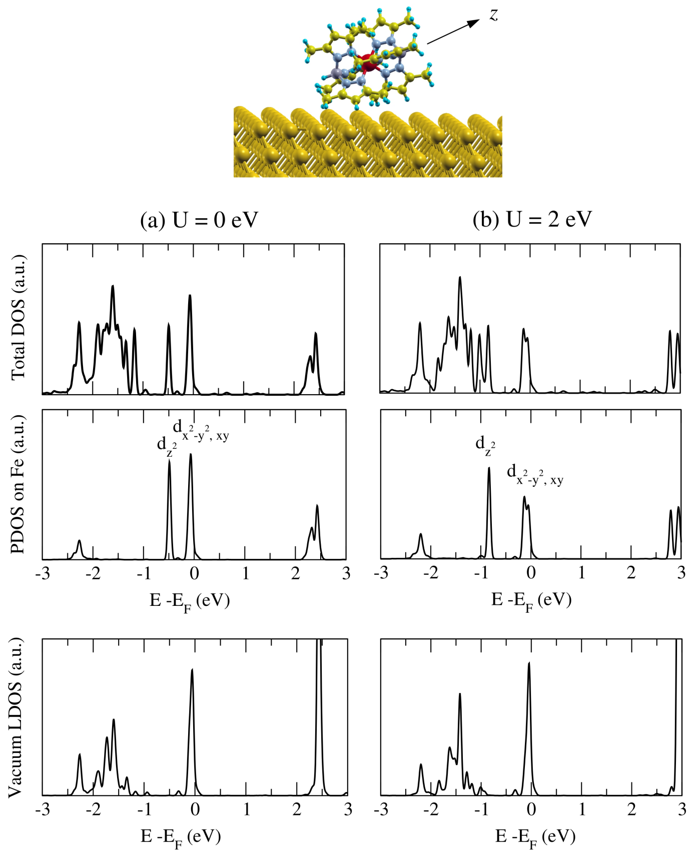

Supplementary Figure 4. **Electronic structure of a LS molecule deposited on a Au(111) surface.** (a) Calculation by DFT. (b) Calculation using DFT+U. (Top panels) Total molecule density of states, (middle panels) PDOS on Fe^{II} atom and (down panels) vacuum local DOS (LDOS) at 5Å above the molecule. Note that we do not show the HS molecule case since it is higher in energy and one would need to do a constraint search calculation by fixing locally magnetic moment on the molecule, the procedure not yet implemented in the Quantum Espresso package. The procedure of fixing the total spin moment (similarly to what has been done for the free molecules) leads to an unphysical solution where Au substrate becomes magnetic when the spin moment is spread over the whole system during the self-consistent run. From the PDOS on Fe^{II} atom, we can clearly see the two-fold degenerate $d_{x^2-y^2}$,d_{xy}-states corresponding to the 2-fold degenerate HOMO and the d_{z^2}-derived HOMO-1 state. Out of these states only the HOMO spreads enough out of the molecule to the vacuum and can thus be probed by the STM tip, the HOMO-1 state being strongly localized inside the molecule (almost no hybridization with the ligand cage) should not be imaged by STM.

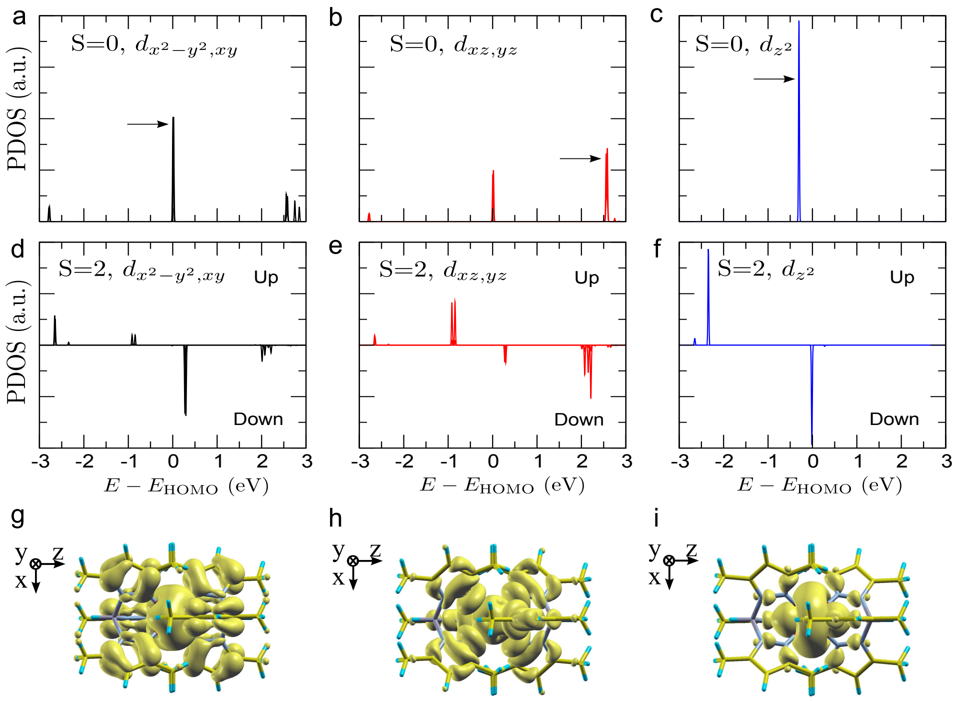

Supplementary Figure 5. **Projected densities of state and isodensities.** Projected density of state on the central Fe^{II} atom for **1** in LS ($S = 0$) and in HS ($S = 2$) states and isodensity for the different d-orbitals. (a) $S = 0$, $d_{x^2-y^2}$,d_{xy}. (b) $S = 0$, d_{xz},d_{yz}. (c) $S = 0$, d_{z^2}. (d) $S = 2$, $d_{x^2-y^2}$,d_{xy}. (e) $S = 2$, d_{xz},d_{yz}. (f) $S = 2$, d_{z^2}. (g), (h), (i) Isodensities of the states indicated by the arrow in (a), (b), (c) respectively. The z-axis is taken along the B-Fe-B axis.

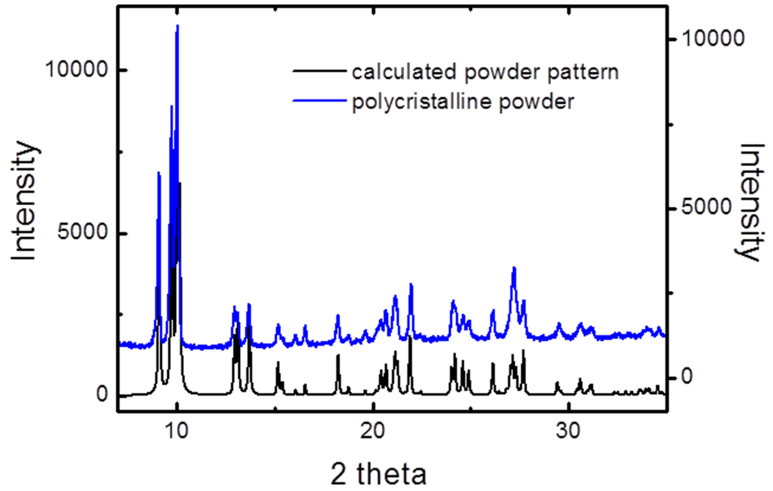

Supplementary Figure 6. **X-ray diffraction data.** Superimposition of the experimental powder X-ray diffractogram of **1** at 289 K with the one calculated from the single crystal X-ray data (289 K)

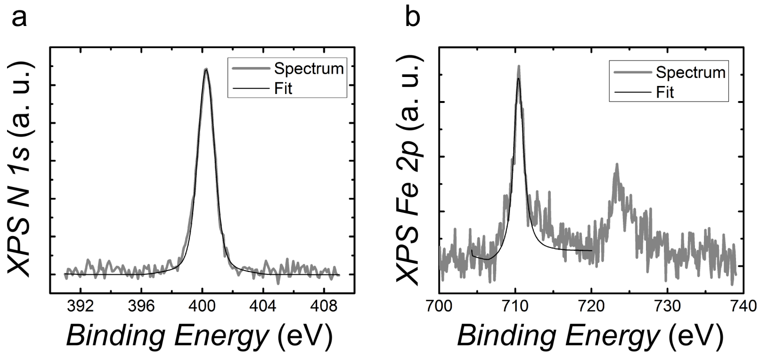

Supplementary Figure 7. **X-ray Photoelectron Spectroscopy of a monolayer.** (a) XPS spectrum in the N 1s region and (b) XPS spectrum in the Fe 2p region recorded on a monolayer of compound **1** on a Au(111)/Mica sample. X-ray Photoelectron Spectroscopy analysis were carried out with a ThermoFisher Escalab 250 XI spectrometer using a monochromatic X-ray Al-kα source. The analysis zone consisted in a 900 μm in diameter spot. The instrument was calibrated in energy with the silver Fermi-level (0 eV) and the $3d_{5/2}$ core level of metallic silver (368.3 eV). The C-1s signal was used to correct a possible charge effect: the C-C/C-H contribution of C-1s spectra was fixed at 285.0 eV. The data processing was performed using the commercial Avantage Software, which allowed the estimation of Fe/N atomic ratio of 11.5 which is in good agreement with the expected value for compound **1**.
